# Radical flanks of social movements can increase support for moderate factions

**DOI:** 10.1093/pnasnexus/pgac110

**Published:** 2022-08-04

**Authors:** Brent Simpson, Robb Willer, Matthew Feinberg

**Affiliations:** Department of Sociology, University of South Carolina, 911 Pickens St, Columbia, SC 29208, USA; Department of Sociology, Stanford University, 450 Jane Stanford Way Building 120, Room 160 Stanford, CA 94305-2047, USA; Rotman School of Management, University of Toronto, 105 St George St, Toronto, ON M5S3E6, Canada

**Keywords:** radical flanks, protest tactics, violent protests, collective action; social movements

## Abstract

Social movements are critical agents of social change, but are rarely monolithic. Instead, movements are often made up of distinct factions with unique agendas and tactics, and there is little scientific consensus on when these factions may complement—or impede—one another’s influence. One central debate concerns whether radical flanks within a movement *increase* support for more moderate factions within the same movement by making the moderate faction seem more reasonable—or *reduce* support for moderate factions by making the entire movement seem unreasonable. Results of two online experiments conducted with diverse samples (*N* = 2,772), including a study of the animal rights movement and a preregistered study of the climate movement, show that the presence of a radical flank increases support for a moderate faction within the same movement. Further, it is the use of radical *tactics*, such as property destruction or violence, rather than a radical *agenda*, that drives this effect. Results indicate the effect owes to a contrast effect: Use of radical tactics by one flank led the more moderate faction to appear less radical, even though all characteristics of the moderate faction were held constant. This perception led participants to identify more with and, in turn, express greater support for the more moderate faction. These results suggest that activist groups that employ unpopular tactics can increase support for other groups within the same movement, pointing to a hidden way in which movement factions are complementary, despite pursuing divergent approaches to social change.

Significance Statement Recent research shows that use of radical tactics, such as violence or property destruction, by activist groups typically decreases support for the activists and can also reduce support for their agenda. But here we provide causal evidence that the use of radical tactics by one movement faction can increase support for moderate factions within the same movement. We show that this “positive radical flank effect” occurs because the tactics employed by the radical flank leads the public to view the more moderate faction’s tactics as less radical in contrast. These results reveal a subtle way in which unpopular tactics of a radical flank can increase public support for moderate factions within the same movement.

Social movements are major drivers of cultural change and social progress (1, 2). There is considerable interest in how social movements can most effectively resonate with audiences to shift public opinion (3–5). Recent studies of “extreme” or radical tactics show that movements whose activists threaten violence or property destruction are generally less successful at winning public support than movements that do not (6–10). Yet, social movements are not homogeneous in the agendas they pursue nor in the tactics they employ (11, 12). Instead, they are typically composed of an array of factions with varying agendas and employing diverse tactics. A critical question is how this diversity within movements impacts the success of any given activist group.

The literature on “radical flanks” addresses this question by asking how more radical factions impact the success of more moderate factions within the same movement, offering competing predictions about the direction of this impact (11–14). The *positive radical flank effect hypothesis* predicts that the presence of a radical flank—a discrete activist group within a larger movement that adopts an agenda and/or uses tactics that are perceptibly more radical than other groups within the movement—will increase support for a more moderate movement faction. The *negative radical flank effect hypothesis* predicts radical flanks will decrease support for more moderate factions within the same movement.

Although there is little empirical support for negative radical flank effects, a number of correlational studies support the positive radical flank effect hypothesis (11, 13). But other empirical tests find no evidence that radical flanks increase or decrease support for moderate factions within the movement (14). Thus, the radical flanks literature has yielded inconsistent findings.

One reason for inconsistencies in prior work may be that observational data on radical flank effects cannot generally distinguish between radical flanks as causes or consequences of variation in public support or success of intra-movement factions. For instance, flanks may turn to radical tactics as a last-ditch effort to call attention to their cause by distinguishing themselves from a moderate faction who is gaining an increasing share of public attention and support (15). In the cross section, this may give the appearance that radical flanks caused—rather than resulted from—the increased success of a moderate faction. Similarly, a more moderate faction may adopt a more popular agenda or tactics to distinguish itself more explicitly from an unpopular radical flank. For these reasons, as Chenoweth and Schock note, “social movement research on the radical flank effect tends to reflect biases of case selection and context” (14).

Inconsistent findings in prior work may also result from broader movement dynamics that moderate whether radical flanks positively or negatively impact support for their moderate counterparts (12). For example, a moderate faction may seek to explicitly disassociate itself from a radical flank in an effort to increase public support and, if successful, create positive radical flank effects. On the other hand, leaders of counter-movements may seek to explicitly associate the moderate faction with its more radical counterparts in order to reduce support for it, negating any positive radical flank effects and perhaps, in the extreme, producing negative flank effects.

Our goal in this research is not to fully capture these complex broader dynamics. Rather, we aim to complement the rich accounts of radical flanks and movement heterogeneity from real-world contexts with results of two controlled survey-experiments conducted on large, diverse samples. Our studies were designed to answer three interrelated questions about whether, when, and why radical flanks impact public support for more moderate factions within a movement. First, we ask whether the presence of a radical flank increases or decreases identification with and support for more moderate movement factions. Public support is crucial to the success of social movement groups (16, 17) and is not just an important end in itself. It is also key to changes in laws and institutions with “protesters first winning public support and public support subsequently affecting politics” (17); see also (18–20). Of course, movement groups may also bring about change via other means, like applying direct pressure to institutions and governments (21–24). But we focus explicitly on how movement groups impact public support, given its direct and indirect roles in driving social change (25).

Recent studies show that the use of radical tactics, such as property destruction or violence, leads to lower support for the particular group or faction employing radical tactics (6–8, 10, 26). According to the negative radical flank hypothesis, these negative effects will spill over to moderate factions within the movement, leading to lower levels of identification and support. The positive radical flank hypothesis predicts instead that the presence of radical flanks will lead to greater identification with and support for more moderate movement factions.

In terms of their hypothesized effects on public perceptions, negative and positive radical flank effects parallel assimilation and contrast effects long studied by psychologists (27, 28). Negative radical flank effects involve assimilation, whereby exposure to a radical flank within a given movement (negatively) impacts perceptions of the broader movement, leading to more negative perceptions of moderate movement factions. On the other hand, a radical flank may provide a negative standard against which other factions within the movement are more favorably judged, leading to contrast—or positive radical flank—effects.

In addition to testing whether radical flanks have positive or negative effects on moderate factions within a given movement, we offer a test of *why* radical flank effects occur. From the perspective of the positive radical flank effect hypothesis, radical flanks present an alternative—by definition, a more radical one—to a moderate movement faction. This salient exemplar will lead the public to view the moderate faction’s tactics or agenda as less extreme or radical by contrast. The rival negative radical flank hypothesis suggests that negative views of radical flanks will spread to the moderate faction. These assimilation effects will lead the moderate faction to be perceived as more extreme or radical than they would have otherwise, a “guilt by association” effect.

We expect that the resulting positive or negative views of moderate factions will lead to changes in identification with—and support for—those factions. That is, since people tend to view themselves as rational and reasonable (29) and given that similarity is a key basis of identification (30, 31), the positive radical flank effect hypothesis predicts that favorable perceptions of a given moderate faction will lead to higher levels of identification with that faction. The negative flank hypothesis, on the other hand, predicts that the negative perceptions of the moderate faction will lead to lower identification. We expect that the higher (for the positive flank effect hypothesis) or lower (negative flank effect) level of identification will drive support for the activist group (8, 32, 33).

While prior work, including naturalistic studies (34, 35), controlled experiments (8, 26), and a meta-analysis (33), suggests that identification is the primary cause of support for movement factions, we also test a more exploratory pathway through which radical flanks may alter support for more moderate factions, namely via changes in perceived normative support for the moderate faction. Specifically, building on theory and evidence of the powerful impact of perceived social norms on behavior (36), recent research suggests that the perception that others support a given movement increases personal engagement with the movement (37). Thus, applied to heterogeneity within movements, if people tend to think that others tend to disapprove of radical tactics agendas of one movement faction they may, in turn, think that others will be more supportive of more moderate factions within the same movement. This greater perceived normative support of a moderate faction would then increase personal support for the moderate movement faction.

Finally, we aim to identify what it is about radical flanks that impacts other factions. Flanks may be radical with respect to their agendas, their tactics, or both. Because radical flanks typically feature both more radical tactics and agendas, prior research has not attempted to disentangle their respective effects (38, 39). Nonetheless, whether radical flank effects primarily occur when flanks are radical with respect to agenda or tactics is important to understand, not only for scholars, but also for movement factions thinking strategically about the impact of tactical decisions. By isolating the effects of tactics and agendas, we aim to better establish when radical flanks will alter public support for a given movement group.

## Empirical Strategy

To answer these questions, we conducted two web-based survey-experiments (see the “Methods” section). Our first experiment investigated radical flank effects in the context of the animal rights movement (40, 41). Our second, preregistered, experiment sought to replicate and extend Study 1 in the context of the climate movement (42, 43). Participants in both studies were told that we were interested in people’s attitudes about different social movement organizations, and that they would be reading information on these organizations “activities and mission statements taken from their official websites and other materials, and from media coverage of the organizations.” Both studies presented participants with two movement factions. First, they learned about the *treatment faction*. We manipulated whether the treatment faction had (i) a radical or moderate agenda and (ii) used radical or moderate tactics. We then measured participants’ perceptions of the radicalness of the treatment faction’s tactics and agenda, perceived normative support for faction’s agenda, identification with the faction, and participant’s support for the treatment faction.

Then participants read about the *focal faction*. The description of the focal faction was identical for all participants. After reading about the focal faction, we measured participants’ perceptions of the radicalness of the focal faction’s tactics and agenda, perceived normative support for its agenda, identification with the faction, and participant’s support for the focal faction. Experiment 2 also included measures of perceived normative support for each faction’s tactics, and measures of participants’ stated willingness to act on behalf of each faction, namely whether they would be willing to attend an event or sign a petition sponsored by the faction.

Although our studies allow for the emergence of either positive or negative radical flank effects, they may be more favorable to finding positive radical flank—or contrast—effects. This is because participants in our studies were exposed to two different movement factions close in time. These conditions may be more apt to generate contrast effects, such that observers will view a more moderate movement faction more favorably than they would have had more time elapsed between exposure to a radical flank and a more moderate movement faction. With a larger time period between exposure to two different factions, observers may be more apt to assimilate information about the initially observed faction (e.g. its tactics or agenda) into perceptions of the faction they observe later. The Discussion addresses these and related dynamics that could moderate our key findings.

Additionally, both studies measured, rather than manipulated, the proposed mediating variables. Thus, our analyses do not allow us to rule out the possibility that participants, for instance, identify with a given faction *because* they support it, rather than support the faction because they identify with it, as we hypothesize. This limitation is important to keep in mind and prior work suggests that the link between identification and support is, to some extent, bidirectional (35). That said, a large literature (8, 26, 34, 35), including a meta-analysis (33) provides a stronger foundation for expecting that identification with a movement faction or protest group leads to increased support for it, rather than the reverse. Indeed, one longitudinal study (35) found that, while the relationship between identification with a movement and support for it was bidirectional, the path from identification to support was stronger.

## Results

### Experiment 1

Our first experiment studied radical flank effects in the context of the animal rights movement. Participants in the moderate agenda condition read about a treatment faction called “Americans Against Animal Cruelty (AAAC),” which they were told seeks to improve the conditions in factory farms while also encouraging Americans to reduce their meat consumption and to buy meat and meat products from “certified cruelty free farms.” In the radical agenda condition, participants read about a treatment faction called “No Animals for Food (NAFF),” which was described as seeking to “completely end human consumption of animals and the consumption or use of all animal byproducts.” (See SI for full text of all manipulations as well as the focal faction description.) Manipulation checks show that participants viewed the treatment faction’s agenda as more radical in the radical agenda condition (independent sample *t*-test: *M* = 5.48 versus *M* = 3.18, *t* = 19.63, *df* = 1,115, *P* < 0.001).

Each treatment faction’s agenda was accompanied by an explanation of its tactics. Activists in the moderate tactics conditions were described as staging peaceful demonstrations and marches around cities, and organizing teach-ins. In the radical tactics condition, participants read that activists in the treatment faction had blocked traffic and prevented entry into the offices of meat producers, and “doused streets and meat delivery trucks with the blood and entrails of animals slaughtered in factory farms” and in some cases advocated violence against animal farmers or meat producers. This manipulation of the radicalness of tactics was effective (*M* = 5.77 versus *M* = 2.43, *t* = 46.09, *df* = 1,115, *P* < 0.001). In line with past research (6–8, 10), participants identified with (*M* = 2.08 versus 3.37, *t* = 13.03, *df* = 1,115, *P* < 0.001) and supported (*M* = 2.53 versus 4.46 *t* = 18.52, *df* = 1,115, *P* < 0.001) the treatment faction less when it pursued a radical—versus moderate—agenda. Participants also identified with (*M* = 2.30 versus 3.11, *t* = 7.83, *df* = 1,115, *P* < 0.001) and supported (*M* = 2.99 versus 3.94, *t* = 8.25, *df* = 1,115, *P* < 0.001) the treatment faction less when it employed radical, versus moderate, tactics. Supplementary Table S3 reports full results for treatment faction outcomes.

After reading about and responding to our measures of the treatment faction, all participants read a description of the focal faction, which was identical across conditions. The focal faction was called “People Against Cruelty to Animals (PACA),” which aimed to “raise public awareness of . . . inhumane treatment of animals raised for food” by staging “demonstrations outside factory farms, the headquarters of global meat producers, and in public spaces.” The description noted that the demonstrations generally involve singing and chanting, and speakers who discuss current living conditions of animals raised for food and how citizens can put pressure on their representatives to change these conditions. Our main focus is on how the treatment faction’s tactics and agenda shape perceptions of, identification with, and support for the focal PACA.

As shown in the ANOVA results reported in Table 1, our manipulation of the treatment faction’s tactics consistently impacted our mediators and key outcomes for focal factions. While perceived normative support for the focal faction’s agenda (*P* = 0.002) and identification with the focal faction significantly varied with the treatment faction’s agenda (*P* = 0.011), we did not find any other effects for the agenda manipulation, nor did we find any interaction between our agenda and tactics manipulation. Analyses reported in the SI (Supplementary Table S5) show that the political party identification does not moderate these findings. Given the weak effects of the treatment faction agenda and the absence of moderation by politics, we turn to planned contrasts for the tactics manipulation.

**Table 1.** Effect of treatment faction’s tactics (moderate or radical) and agenda (moderate or radical) on perceptions of and support for the focal faction. Experiment 1.

	**Means (SDs) by condition**				**ANOVA results**		
	**Mod. tactics mod. agenda**	**Rad. tactics mod. agenda**	**Mod. tactics rad. agenda**	**Rad. tactics rad. agenda**	**Radical tactics**	**Radical agenda**	**Interaction**
Perceived radicalness of focal faction tactics	2.38 (1.05)	2.16 (1.03)	2.19 (1.08)	2.14 (1.00)	*F* = 4.81 *P* = 0.029	*F* = 2.74 *P* = 0.098	*F* = 2.13 *P* = 0.145
Perceived radicalness of focal faction agenda	2.20 (1.45)	2.03 (1.33)	2.15 (1.51)	1.99 (1.29)	*F* = 3.80 *P* = 0.051	*F* = 0.37 *P* = 0.543	*F* = 0.01 *P* = 0.934
Identify with focal faction	3.89 (1.84)	4.36 (1.76)	4.28 (1.82)	4.52 (1.76)	*F* = 10.99 *P* < 0.01	*F* = 6.47 *P* = 0.011	*F* = 1.13 *P* = 0.288
Support for focal faction	4.90 (1.65)	5.32 (1.60)	5.12 (1.78)	5.17 (1.75)	*F* = 5.50 *P* = 0.019	*F* = 0.13 *P* = 0.717	*F* = 3.43 *P* = 0.064

The *t*-tests given in Table 2 show that when the treatment flank employed radical tactics, participants viewed the focal faction’s tactics and agenda as less radical than when the treatment flank employed moderate tactics (*P* ≤ 0.05). Participants also identified more with the focal faction when the treatment faction employed more radical tactics (*P* ≤ 0.001). Most importantly, our first key question centers on how the presence of radical flanks impacts support for the more moderate faction. The positive radical flank hypothesis predicts that a radical flank (in this case, a flank that employs radical tactics) will increase support for the moderate faction while the negative radical flank hypothesis predicts that the existence of a radical flank will decrease support for the focal faction. Consistent with the positive radical flank hypothesis, planned comparisons showed higher levels of support for the focal faction when the treatment faction’s tactics were radical than when they were moderate (*P* ≤ 0.05). In other words, even though all participants read the same information about the focal faction, their level of support for this faction depended on the presence of a treatment faction that employed more radical (versus moderate) tactics. Regression models reported in Supplementary Table S6 show that these results are robust to the inclusion of various demographic controls.

**Table 2.** Effect of radical tactics on perceptions of support for focal factions in experiment 1 (animal rights movement) and experiment 2 (climate movement).

	**Means (SDs) and planned contrasts**					
	**Experiment 1**			**Experiment 2**		
	**Radical tactics**	**Moderate tactics**	***t***	**Radical tactics**	**Moderate tactics**	***t***
Perceived radicalness of tactics	2.15 (1.01)	2.28 (1.07)	2.16*	14.94 (19.64)	19.14 (20.64)	4.28***
Perceived radicalness of agenda	2.01 (1.31)	2.17 (1.48)	1.95*	19.05 (22.30)	23.15 (22.05)	3.76***
Identification moderate faction	4.44 (1.76)	4.09 (1.83)	3.29***	59.02 (30.29)	53.78 (30.21)	3.53***
Support for moderate faction	5.25 (1.68)	5.01 (1.72)	2.30*	73.15 (27.71)	69.31 (27.84)	2.81**
Action intentions moderate faction	*Not measured*			53.13 (32.71)	48.88 (32.78)	2.64**

*P ≤ 0.05; **P ≤ 0.01; ***P ≤ 0.001.

The results, thus far lend clear causal support for the positive radical flank effect hypothesis and against the negative radical flank hypothesis. The fact that we consistently observe effects for radical tactics, but only find effects of a radical agenda manipulation for the identification measure also offers a tentative answer to the question of whether radical flanks impact support for moderate factions due to tactics, agenda, or both. It appears tactics are what really matters.

Why did the use of radical tactics by the treatment faction increase support for the focal faction*?* As outlined above, if results yielded support for the positive radical flank hypothesis, then we would expect that this would be driven by the presence of a radical flank leading the public to view the focal faction as less radical, thus leading to greater identification with and support for the focal faction. To test this explanation, we ran a series of mediation models given in Supplementary Figure S1. [Supplementary Table S7 contains sensitivity analyses (44, 45) for these mediation models, as well those presented for Study 2.]

First, we assessed the mediating role of perceived radicalness of the focal faction’s tactics on support for the focal faction. Bootstrap mediation analyses (46) show that perceived radicalness of the focal faction fully mediated the effect of the radical tactics manipulation on support for the focal faction (Supplementary Figure S1A; CI [.01, 0.19]). We then tested whether radical tactics by the treatment flank would lead to greater support for the focal faction via enhanced identification with the focal faction. Identification with the focal faction fully mediated the impact of radical tactics by the treatment group on support for the focal faction (Supplementary Figure S1B; CI [.11, 0.43]). Finally, we conducted a serial mediation analysis to assess the full explanatory path whereby radical tactics by the treatment flank leads the public to view the focal faction’s tactics as less radical, which in turn leads to higher levels of identification with them and thus more support for them. While the two indirect paths via perceived radicalness of the focal faction’s tactics and identification with the focal faction continued to act as mediators, we also find evidence for the serial path through perceptions of the radicalness of the focal faction tactics and identification with the focal faction (Supplementary Figure S1C; CI [.01, 0.10]).

Although it was not a primary aim of our research, it is important to know whether radical flanks increased support for the focal faction without undermining support for broader movement issues, i.e. objectives beyond the specific agendas of the movement factions we investigated. To address this, participants were asked about broader animal rights issues, e.g. “How likely is it that you will reduce your consumption of meat (e.g. by taking part in ‘Meatless Mondays’).” (See the SI for all measures.) Our radical tactics manipulation did not impact responses to any of these measures, suggesting that the presence of flanks who use more radical tactics can boost support and willingness to act on behalf of the focal faction without harming more general support for the movement.

### Experiment 2

We conducted a second experiment in the context of the climate movement. Experiment 2 featured several improvements on our first study, including better measures of perceived radicalness of tactics and agendas (see Supplementary Table S1), a measure of perceived normative support for each faction (see Supplementary Table S2), an additional measure of personal support, and a larger sample size (see the “Methods” section). Additionally, in Study 1, the name of the treatment faction differed in the radical and moderate agenda conditions. While it seems unlikely that this would have influenced our results, experiment 2 holds the name of the treatment faction constant across conditions. Finally, we preregistered the sampling plan, analyses, and several key hypotheses for experiment 2 (main preregistration file is here: OSF Registries | Radical Flank Environmental Study Fall 2020; an amendment, submitted prior to data collection, with improved exclusion criteria is given here: https://osf.io/yqv72.)

Given the debate in the literature regarding whether radical flanks have positive or negative effects, we preregistered both positive and negative radical flank effect hypotheses on identification and support for the focal faction. Similarly, we preregistered hypotheses for both radical tactics and a radical agenda. But the results of experiment 1 suggest we should be more likely to observe positive radical flank effects and that these effects will be driven by radical tactics by the treatment faction, rather than a radical agenda. We also preregistered two different (and nonmutually exclusive) mediation models, one where identification with the focal faction mediates the effect of a radical flank on support for the focal faction, and one where perceived normative support for the focal faction mediates the effect of a radical flank on support for the focal faction.

All Study 2 participants read about a treatment faction called “Climate Action Today,” but the tactics and agenda of the group varied by experimental condition. In the moderate agenda condition, Climate Action Today was described as seeking to phase out “the use of fossil fuels for automobiles and heating systems in homes and other buildings over the next 15 years.” In the radical agenda condition, it was instead described as seeking “an immediate end to the use of all fossil fuels, including fuel for automobiles and homes and other buildings, within a year.” Manipulation checks show that treatment faction agenda was perceived as more radical in the radical agenda condition (*M* = 66.07) compared to the moderate agenda condition (independent samples *t*-test: *M* = 40.02, *t* = 19.03, *df* = 1,654, *P* < 0.001).

The radical versus moderate tactics manipulation was similar to the one used in experiment 1. In the moderate tactics condition, Climate Action Today activists were described as having organized “peaceful marches” and sponsored “mass teach-ins” to educate the public about climate change and the need to address it. In the radical tactics condition, activists had damaged the property of fossil fuel companies, and threw stones and bottles at the cars of their employees. Manipulation checks showed that participants viewed the faction’s tactics as more radical in the radical (*M* = 72.37) versus moderate tactics condition (*M* = 25.89, *t* = 42.04, *df* = 1,654, *P* < 0.001).

ANOVA results for the treatment factions are reported in Supplementary Table S4. Consistent with Study 1 and prior research (6–10), we find strong negative effects of both radical tactics and radical agendas on identification with (*P* ≤ 0.001) and support (*P* ≤ 0.001) for the treatment faction. We also find strong negative effects of agenda and tactics on stated willingness to act on behalf of the treatment faction (*P* ≤ 0.001).

Participants then read about the focal faction called Global Warming Warning, whose tactics and agenda were held constant. Global Warming Warning was described as focused on educating the public about the scientific consensus regarding the causes of global warming, and communicating the urgency of addressing climate change. Further, they were described as using scientific research to develop best practices for specific ecosystems and communities. They pursued this agenda by lobbying state and national governments, staging peaceful demonstrations in front of government agencies and other public spaces, sponsoring petitions, and organizing local training programs to educate local stakeholders about how to best manage and improve infrastructure and natural resources. Again, we ask how variation in the radicalness of the treatment faction’s tactics and agenda impacted participant’s perceptions, identification with and support for this focal faction. We also ask how radical tactics and agenda impact perceived normative support for the focal faction and willingness to act on behalf of the focal faction.

The ANOVA results for the focal faction are given in Table 3. The stronger effects of the treatment faction’s tactics (versus agenda) on perceptions of the focal faction we observed in experiment 1 are even clearer in experiment 2. Indeed, while radical tactics by the treatment faction significantly impacted every outcome of the focal faction, the agenda manipulation had no significant effects on any outcome for the focal faction. This is additional evidence for our conclusion from experiment 1 that radical flank effects occur when the flank uses radical tactics more than when it has a radical agenda. The SI also investigates whether any of our key findings are moderated by political party identification (Supplementary Table S5). As in Study 1, they are not. We return to these points later and, following our preregistered analysis plan, set aside our agenda manipulation and instead focus the remainder of our analyses on how treatment faction tactics impacted perceptions of and support for focal factions.

**Table 3.** Effect of treatment faction’s tactics (moderate or radical) and agenda (moderate or radical) on perceptions of and support for the focal faction. Experiment 2.

	**Means (SDs) by condition**				**ANOVA results**		
	**Mod. tactics mod. agenda**	**Rad. tactics mod. agenda**	**Mod. tactics rad. agenda**	**Rad. tactics rad. agenda**	**Radical tactics**	**Radical agenda**	**Interaction**
Perceived radicalness of focal faction tactics	19.57 (20.96)	15.15 (19.45)	18.70 (19.56)	14.75 (19.84)	*F* = 18.18 *P* < 0.001	*F* = 0.041 *P* = 0.520	*F* = 0.06 *P* = 0.809
Perceived radicalness of focal faction agenda	23.42 (23.27)	19.81 (22.99)	22.87 (20.77)	18.32 (21.63)	*F* = 13.98 *P* < 0.001	*F* = 0.89 *P* = 0.347	*F* = 0.18 *P* = 0.669
Identify with focal faction	52.55 (31.39)	58.19 (30.15)	55.01 (28.94)	59.81 (30.43)	*F* = 12.29 *P* < 0.001	*F* = 1.89 *P* = 0.170	*F* = 0.08 *P* = 0.778
Support for Focal Faction	68.44 (28.64)	73.52 (27.38)	70.19 (27.01)	72.80 (28.04)	*F* = 7.92 *P* = 0.005	*F* = 0.14 *P* = 0.707	*F* = 0.82 *P* = 0.366
Willingness to act on behalf of focal faction	48.62 (33.40)	53.44 (32.08)	49.14 (32.17)	52.85 (33.33)	*F* = 6.99 *P* = 0.008	*F* = 0.00 *P* = 0.984	*F* = 0.12 *P* = 0.730

Table 2 presents *t*-tests of the effects of radical tactics by the treatment faction on outcomes for the focal faction. These results show that the use of radical (versus moderate) tactics by the treatment faction led participants to view the focal faction as having a less radical agenda and as using less radical tactics, and also led to greater perceived normative support for the focal faction (*P* ≤ 0.001). Similarly, we find strong support for the preregistered prediction that the presence of radical tactics by the treatment faction will lead to higher levels of identification with the focal faction (*P* ≤ 0.001). Most importantly, in line with experiment 1 and our preregistered prediction for radical tactics, we find that participants in the radical tactics treatment condition reported higher levels of support for the focal faction than did those in the moderate tracts treatment condition (*P* ≤ 0.01). Experiment 2 also included an additional indicator of support: stated willingness to act on behalf of the focal faction. Adding further support to the positive radical flank hypothesis, those in the radical tactics condition expressed greater willingness to act on behalf of the focal faction than did those in the moderate tactics condition (*P* ≤ 0.01).

**Fig. 1.**
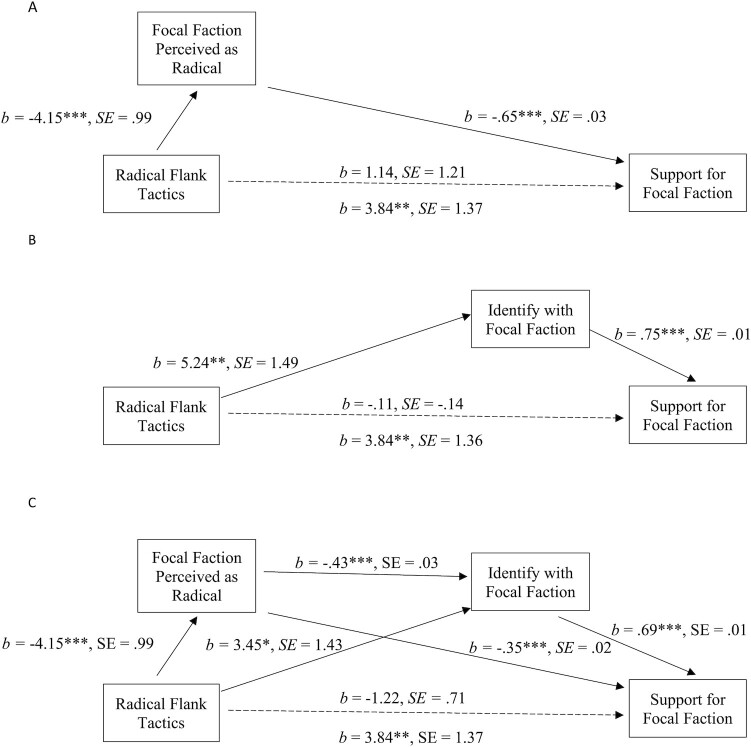
Mediation model for *Support for the Focal Faction*. Indirect path: CI [1.41, 4.03] (A). Mediation model for *Support for the Focal Faction*. Indirect path: CI [1.78, 6.12] (B). Serial mediation model for *Support for the Focal Faction*. Serial path: CI [.61, 1.92] (C). * *P* ≤ 0.05; ** *P* ≤ 0.01; *** *P* ≤ 0.001.

**Fig. 2.**
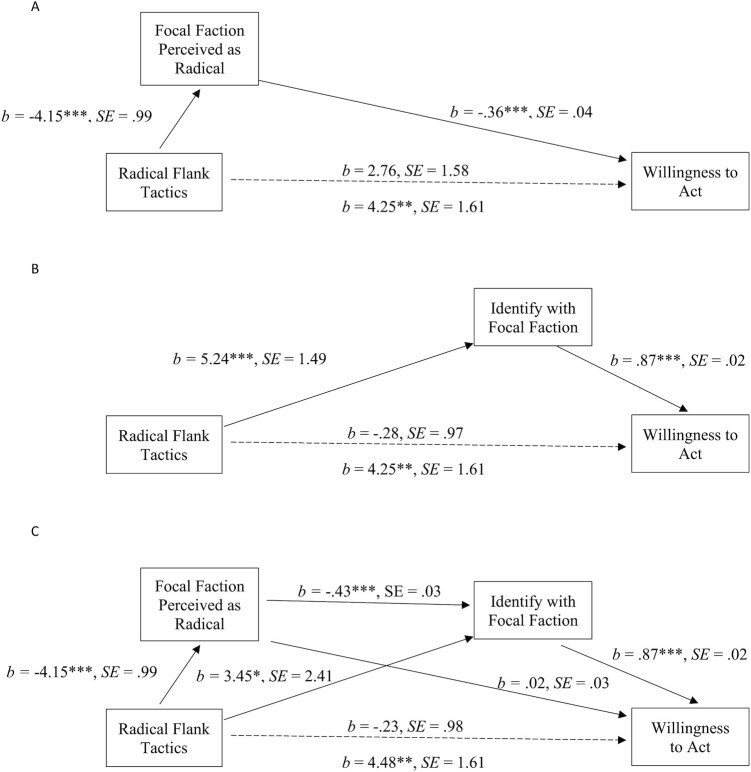
Mediation model for *Willingness to Act on Behalf of the Focal Faction*. Indirect path: CI [.75, 2.38] (A). Mediation model for *Willingness to Act on Behalf of the Focal Faction*. Indirect path: CI [2.06, 7.07] (B). Serial mediation model for *Willingness to Act on Behalf of the Focal Faction*. Serial path: CI [.80, 2.42] (C). * *P* ≤ 0.05; ** *P* ≤ 0.01; *** *P* ≤ 0.001.

Again, we ask why the use of radical tactics by a movement flank (i.e. the treatment faction) increased support for—and willingness to act on behalf of—the moderate faction. First, we assess the mediating role of perceived radicalness of the focal faction’s tactics on our two key outcomes. Bootstrap mediation analyses show that perceived radicalness of the focal faction fully mediated the effect of the radical tactics manipulation on support for the focal faction (Figure 1A; CI [1.41, 4.03]) and willingness to act on its behalf (Figure 2A; CI [.75, 2.38). We also tested whether radical tactics by the treatment flank would lead to greater support for—and willingness to act on behalf of—the focal faction via enhanced identification with the focal faction. Identification with the focal faction fully mediated the impact of radical tactics by the treatment faction on support for the focal faction (Figure 1B; CI [1.78, 6.12]) and willingness to act on its behalf (Figure 2B; CI [2.06, 7.07]).

Given these results, we again tested the full explanatory path that we tested in experiment 1, whereby radical tactics by the treatment flank lead the public to view the focal faction’s tactics as less radical, which in turn leads to higher levels of identification with them and thus more support for and willingness to act on behalf of the focal faction. For the support outcome, while the two indirect paths via perceived radicalness of the focal faction’s tactics and identification with the focal faction continued to act as mediators, we also find evidence for the serial path through perceptions of the radicalness of the focal faction tactics and identification with the focal faction (Figure 1C; CI [.61, 1.92]). We also find support for the serial mediation through perceived radicalness of the focal faction and then through identification with the focal faction (Figure 2C; CI [.80, 2.42]) for willingness to act on behalf of the focal faction.

As noted above, prior work suggests that identification is the most important predictor of support for activist groups and social movement factions. And the results thus far lend support to the claim that identification also plays a causal role in radical flank effects. But we also explored another potential mediator, namely perceived normative support. We caution that it is difficult to know the extent to which perceived normative support is causing—versus being caused by—personal support. For instance, it may that participants “project” their support for a given faction onto others, assuming that others’ support falls in line with their own. And, in contrast to the link between identification and personal support, existing theory and empirical work does not give us a strong basis for expecting that one causal direction will be stronger than the other in the link between perceived normative support and personal support. Thus, these models should be taken as suggestive.

Mediation analyses given in Supplementary Figure S2 show that perceived normative support mediates the effect of radical tactics by the treatment faction on support for the focal faction (CI [5.50, 9.05]). In fact, Supplementary Figure S2A shows that, once we account for this mediation effect, the direct effect of radical tactics by the treatment faction become negative, an issue we take up in the Discussion section. Mediation analyses presented in Supplementary Figure S2B show that perceived normative support for the focal faction fully mediates the effects of treatment faction tactics on willingness to act on behalf of the focal faction (CI [4.99, 8.51]). Unlike the *support* outcome of Supplementary Figure S2A, we do not find any evidence of a suppressor effect for the willingness to act outcome measure.

Given these findings, we conducted parallel mediation models to simultaneously test for perceived normative support and identification with the focal faction as mediators for both our support measure and willingness to act measure. These results, the full details of which are on the OSF page for this project (https://osf.io/4zav8/), show that both perceived normative support and identification with the focal faction explain the impact of radical faction tactics on our two key outcome measures.

First, for analyses of the support measure, bootstrap confidence intervals for the contrast between the indirect path through perceived normative support and indirect path through identification included 0 (CI −1. 50, 1.59), suggesting that neither path is a significantly stronger mediator of the use of radical tactics by the treatment faction on support for the focal faction (3). However, for the *willingness to act* outcome, the confidence interval did not include 0 (CI −5.15, −0.59), indicating that the path through identification with the focal faction plays a stronger role. Based on these analyses, we conclude that while perceptions of normative support may play a (largely independent) role in driving support, identification appears to play a more robust mediating role.

Finally, as in our first study, we wanted to ensure that the presence of a radical flank increased support for—and willingness to act on behalf of—a more moderate faction without undermining support for broader movement objectives, i.e. beyond the specific activists and agendas of the climate movement factions we investigated. To assess this, we included items adapted from an environmental citizenship measure (47) and measures of climate policy support (48). (See SI for all items.) As in Study 1, we did not find any evidence that participants in the radical tactics condition scored lower on any of the environmental citizenship or climate policy support measures suggesting, again, that the use of radical tactics by one movement faction can boost support and willingness to act on behalf of a more moderate focal faction without harming support for the movement in general.

## Discussion

Research on radical flanks provides intuitively appealing descriptive accounts of observational data on social movements. But this literature has also been characterized by persistent ambiguities (12, 14). Here, we brought causal evidence to bear on radical flank effects to answer three questions (i) whether the presence of radical flanks can lead to more (or less) public support for moderate factions within a movement, (ii) why such radical flank effects occur, and (iii) whether these effects primarily occur when radical flanks have a radical agenda, employ radical tactics, or both. Answering these questions is helpful for understanding how and when social movements bring about change.

We addressed our first question by studying whether the presence of a radical (versus moderate) flank within the animal rights movement (Study 1) and climate movement (Study 2) can increase or decrease support for a comparatively moderate focal faction, whose tactics and agenda were held constant. Findings from both studies established causal evidence for the positive radical flank hypothesis, demonstrating that the presence of a radical flank can increase identification with and support for a moderate faction in the same social movement.

Both studies also allowed us to assess why radical flanks can increase support for moderate factions. Serial mediation models supported the predicted contrast effect whereby the presence of a radical, versus moderate, flank led the same focal faction to be viewed as less extreme. These more favorable perceptions led to higher levels of identification with the focal faction which, in turn, increased support for—and willingness to act on behalf of—the focal faction. Experiment 2 also provided suggestive evidence for a noncompeting mediating mechanism, namely the perception that others tended to support the focal faction.

Finally, both experiments suggest an answer to our third question, whether positive radical flank effects occurred when the radical flank pursued a more radical agenda, when it used radical tactics, or both. Previous research on radical flanks has at least implicitly assumed that flanks are simultaneously radical with respect to agenda and tactics. Yet social movement scholars have called for research that more clearly distinguishes agendas and tactics (38, 39). In both studies, we independently manipulated whether flanks were radical with respect to tactics or agenda. We consistently found that tactics mattered for the emergence of flank effects, but agendas did not. This result may help explain mixed support for the radical flank hypothesis in real world social movements, where flanks vary in the extent to which they are radical with respect to agenda or tactics. Findings from both of our experiments suggest that radical flank effects are more likely when flanks are radical with respect to tactics.

It is important for future research to assess the robustness of our finding that tactics matter more than agendas, but also to understand why this is the case. We suspect that, particularly for members of the general public (as opposed to committed activists within a given movement), actions speak louder than words. Tactics may be seen as more reliable information since they are tangible actions that have actually happened, while agendas are often more carefully crafted for self-presentation purposes. Thus, to someone outside of the animal rights movement, variation in the factions’ agendas may say less about the radicalness of each faction than the tactics they use to pursue those agendas.

Asymmetries in the effects of tactics and agendas would be important to understanding radical flank processes in the real world since observers may, at any given time, have limited information about either the tactics or agenda of a faction. In such cases, observers may tend to infer the tactics (or agenda) of one movement faction from those of another. Future research could address these and related issues by modifying the studies presented here to limit the information participants receive about the tactics and/or agendas of a given faction.

Several other questions are also important for future research. Foremost, as noted earlier, both of our experiments employ procedures that are arguably more favorable to finding positive—versus negative—radical flank effects. Specifically, our study participants learned about treatment and focal factions in relative quick succession. This might have created a stronger contrast between the two factions than would have occurred had information on the two factions been more spaced out over time. With more time between exposure to the treatment and focal factions, participants might have been more likely to assimilate their impressions of the radical treatment faction into their judgments of the focal faction. This should be addressed in future work that manipulates the amount of time that elapses between exposure to different movement factions.

More generally, the studies reported here provide a point of departure for developing a more thorough and dynamic understanding of radical flank effects. For instance, an important next step is to investigate what happens when a wider set of social actors (leaders of counter-movements, political elites, or a highly polarized media) strategically associate or disassociate radical flanks and moderate factions in an effort to alter public support for the moderate faction. In our experiments, the moderate factions did not explicitly disassociate themselves from the radical flanks, which could have strengthened the contrast effects we observed here, thus leading to even stronger positive radical flank effects (13). On the other hand, if actors opposed to the movement can convince the public (accurately or not) that the two factions are actually a single group or are otherwise closely aligned, we might expect an attenuation of positive radical flank effects and, in the extreme, potential negative flank effects. Indeed, conflicting observations in the literature may have resulted from variation in moderate activists’ efforts to disassociate themselves from radical flanks as well as movement opponents’ efforts to strategically connect radical flanks to moderate factions. Future research could address the relative success of competing efforts to enhance and attenuate flank effects, with the goal of developing a model of radical flank effects that explains when we will tend observe positive versus negative flank effects.

That some processes would give rise to negative—rather than positive—radical flank effects is further suggested by the suppression effect we observed in experiment 2 (Supplementary Figure S2). Specifically, after accounting for the mediating effects of perceived normative support, we observed a negative direct effect of radical tactics by the treatment faction on support for the focal faction, suggesting a partial tendency for observers to assimilate information about radical tactics from one movement faction into another. Although this tendency was offset in our study by the more positive effects of identification with the focal faction and perceived normative support for the focal faction, future research should explore what processes might be more likely to lead to stronger assimilation—versus contrast—effects.

Given the possibility of strategic (dis)association effects discussed earlier, our finding that radical flank effects were driven by tactics rather than agenda is important not only for social movement scholars, but also activists. Specifically, this finding suggests that “strategic differentiation” on tactics may be most effective in generating support for more moderate factions within movements. If so, activists from moderate factions may benefit from strategically deploying and calling attention to radical flanks who differ primarily in their tactics to create greater support for the moderate faction of the movement.

The radical flank hypothesis was introduced to explain how the existence of a radical faction within a movement might impact the success of more moderation factions within the same movement (11). But future research should explore whether radical factions can also impact support for factions in overlapping or neighboring movements. The presence of such effects likely depends on what factions observers perceive as belonging to the same social movement category. Given that extensive research shows that the boundaries laypeople apply to real world categories tend to be vague (49), radical flank effects may plausibly extend to factions in adjacent movements (50). For instance, while it seems likely that (as in our research) people will view two different climate movement factions as belonging to the same movement or “category,” it is possible that observers may also tend to view animal rights groups or racial justice groups as overlapping with environmental movements, particularly since some movement factions implicate animal agriculture in climate change and others highlight the differential impact of climate change on racially minoritized groups. If so, radical flank effects may occur more broadly than has been suggested thus far. It is also important to investigate whether the effects identified here also apply outside of social movements. For instance, do more “extreme” factions within a given political party increase or decrease support for more moderate factions within the same party? These questions could be addressed with relatively straightforward extensions of the studies we reported in this paper.

It is notable that political party identification did not moderate any of our key experimental effects. One might have expected that Democrats would have been more likely to perceive and appreciate distinctions between various animal rights or climate movement groups since they are aligned more closely with Democratic party politics. Republicans, on the other hand, would have been more apt to ignore or downplay those distinctions, perhaps even taking the tactics of a radical flank as justification for demonizing the moderate faction. But this is not what we found. While those who identified more as Republicans were, unsurprisingly, less supportive than those who identified more as Democrats of the focal animal rights (Study 1) and climate movement (Study 2) factions, the radical flank effect did not depend on whether the person identified as a Democrat, Republican, or Independent. This provides initial evidence that whether we observe positive or negative radical flank effects does not depend on the extent to which observers identify as Republicans or Democrats. A key implication is that the presence of a radical flank should, all else equal, have a net benefit on support for a moderate faction, even in politically diverse populations.

## Methods

### Experiment 1

#### Ethics statement

Experiment 1 was approved by the Institutional Review Board at Stanford University. Informed consent was obtained from all participants.

#### Participants

Participants were recruited from a survey panel of workers recruited from Amazon Mechanical Turk to complete a large prescreen survey that included demographic measures, and elimination of users who fail screening questions of distinct type or whose IP address are listed as fraudulent on any of several inventories (via APIVoid). To ensure that our findings were robust to political party identification, we recruited an equal mix of Republicans, Independents, and Democrats. We excluded all but the first response from any duplicate IP addresses or Mechanical Turk Worker IDs. Our analyses are based on the remaining *N* = 1,116 responses.

#### Design and procedures

Experiment 1 employed a 2 × 2 between-subjects design. Each participant was exposed to two groups within the animal rights movement, a treatment faction and a focal faction. Participants first read about the treatment faction. We fully crossed whether the treatment faction’s agenda was radical or moderate with whether the treatment faction’s tactics were radical or moderate (see the SI for full text of manipulations). Participants then completed several comprehension check questions about the treatment faction. Thereafter, they answered a number of questions about the treatment faction (see *Measures and Manipulation Checks* below). We are primarily interested in how the presence of a radical flank impacts identification with and support for the focal faction who was always presented as having a comparatively moderate agenda and employing moderate tactics. Thus, all participants read the same description of the focal faction. After reading the description of the focal faction, participants completed several comprehension check questions, and then completed the same measures they completed for the treatment faction, but this time for the focal faction.

#### Measures and manipulation checks

For both the treatment and focal factions, we measured perceptions of the radicalness of their tactics and agendas, perceived normative support of each faction, identification with each faction, and support for the faction. (All measures used 7-point scales. See SI for individual items and scale reliability.) It is possible that having participants answer the same questions about each faction could potentially lead to anchoring effects (51). Note, however, that anchoring would have most likely led participants to report similar ratings of the treatment and focal factions, a pattern consistent with *negative* radical flank effects. The fact that we find a contrast effect suggests anchoring was not an issue in our designs.

### Experiment 2

#### Ethics statement

Experiment 2 was approved by the Institutional Review Board at Stanford University. Informed consent was obtained from all participants.

#### Participants

Participants were recruited from the same panel as experiment 1. Per our preregistration, we aimed for a sample size of around 1,600 and excluded all but the first response from any duplicate IP addresses or Mechanical Turk Worker IDs. There were no other exclusions, giving an analytic sample of 1,656.

#### Design and procedures

Experiment 2 presented participants with two different factions within the climate movement. Otherwise, the study was very similar to experiment 1 with a few exceptions. In addition to the larger sample, we included a number of improved measures (see SI for all manipulations and measures).

Like experiment 1, experiment 2 was a 2 × 2 between-subjects design with similar instructions. Each participant was exposed to two factions within the climate movement, a treatment faction (called “Climate Action Today”) and a focal faction (“Global Warming Warning”). The description of the focal faction’s agenda and tactics were held constant, but the treatment faction’s agenda and tactics were manipulated independently.

#### Measures and manipulation checks

For both the treatment faction and focal faction, we measured perceptions of the radicalness of their tactics and agendas, perceived normative support of each faction, identification with each faction, and support for the faction. We also measured willingness to act on behalf of the focal faction. All measures used 100-point scales. See SI for individual items and scale reliability.

## Supplementary Material

pgac110_Supplemental_Files

## Data Availability

Datasets for both studies reported in this paper are available at the OSF page for this project (https://osf.io/4zav8/).
