# Correction to: Radical flanks of social movements can increase support for moderate factions

**DOI:** 10.1093/pnasnexus/pgad478

**Published:** 2024-01-30

This is a correction to: Brent Simpson, Robb Willer, Matthew Feinberg, Radical flanks of social movements can increase support for moderate factions, *PNAS Nexus*, Volume 1, Issue 3, July 2022, pgac110, https://doi.org/10.1093/pnasnexus/pgac110

During a retroactive audit conducted by *PNAS Nexus*, it was discovered that this paper was missing a statement acknowledging compliance with the *PNAS Nexus* Human and Animal Participants and Clinical Trials policy:

Informed consent was obtained from all participants.

This error has been corrected in the original article.

