# Supplementary material for: Radical flanks of social movements can increase support for moderate factions
Source: PNAS Nexus. 2022 Aug 4;1(3):pgac110. doi: 10.1093/pnasnexus/pgac110 (PMC9896934; doi:10.1093/pnasnexus/pgac110)
Supplement: pgac110_Supplemental_Files [file pgac110_supplemental_files.zip › PNASNEXUS-PNASNEXUS-2021-00138-s01.pdf]

Supplementary Information for
Radical Flanks of Social Movements Can Increase Support for Moderate Factions

Brent Simpson, Robb Willer, and Matthew Feinberg

Corresponding author: Brent Simpson
Email: bts@sc.edu

This PDF file includes:

Supplementary text
Figures S1 and S2
Tables S1 to S7
Full Text of Experimental Manipulations
SI References

Supplementary Information Text

Measures of Perceived Radicalness of Agenda and Tactics

For Experiment 1, we measured perceived radicalness of tactics using the items in Table S1. Applied to the treatment factions, these measures serve as our manipulation checks for our radical tactics and agenda manipulations. Applied to the focal factions, these measures allow tests of the contrast and contagion (or assimilation) effects hypothesized to underlie the positive and negative radical flank effects, respectively.

The scale was highly reliable for the treatment faction (α = .95) and somewhat less so for the focal faction (α = .82). The changes we made to the scale in Experiment 2 resulted in comparably high reliability for treatment (α = .95) and focal (α = .93) factions.

For the agenda measure, in Experiment 1, "would entail a change to the status quo" did not hang together with "extreme" (α = .17 for the treatment faction). Thus, in Experiment 1, our analyses of the perceived extremity of agenda are based on the single item, *extreme*. Our second study added three new items, and resulted in highly reliable scales for both the treatment faction (α = .92) and focal faction (α =.91).

How Radical Tactics and Agendas Impact Perceived Normative Support for Treatment and Focal Factions

We measured whether our manipulations impacted perceived normative support for the treatment and focal factions. We expected that participants would view radical flanks as lacking public support, but that radical flanks would increase perceived normative support for the focal faction. As shown in Table S2, we measured perceived normative support for each group's agenda in Experiments 1 and 2. Experiment 1 did not include measures of perceived normativity of tactics. These items were introduced in Experiment 2. The scale for perceived normative support of each faction's agenda in Experiment 1 was highly reliable (α = .95 for treatment faction; α = .93 for the focal faction). Thus, our analyses are based on a composite of the two items. The items for each faction's agenda in Experiment 2 were slightly adapted to allow for closer parallels between the perceived normativity of tactics and agenda and to allow us to test another pre-registered causal pathway through which radical flanks might increase support for moderate factions, namely perceived normative support for the focal faction.

The results for perceived normative support for focal factions are reported in the main text. These results are based on a *perceived normative support* composite (α = .94),

which includes the two perceived normative support of agenda items and the two perceived normative support of tactics items given in Table S2.

Measures of Identification and Support

To measure identification, participants in both studies were asked: "How much do you identify with [treatment/focal faction] activists?" and "How similar do you feel to [treatment/focal faction] activists?" Reliability was high (α =.95 for Treatment Faction, Experiment 1; α = 95 for Focal Faction, Experiment 1; α = .96 for Treatment Faction, Experiment 2, and α = .96 for Focal Faction, Experiment 2). Thus, we composited the two items.

For our measures of support, participants in Experiment 1 were asked: i) "How much do you support the [treatment/focal faction] activists?" and ii) "How much do you support the goals and objectives of [treatment/focal faction]. Participants in Experiment 2 were asked those same questions and one more: "How much do you support the activities of [treatment/focal faction]?" Again, reliability was high (α = .90 for Treatment Faction, Experiment 1; α = .95 for Focal Faction Experiment 1; α = .89 for Treatment Faction Experiment 2; and α = .96 for Focal Faction, Experiment 2). Thus, our analyses are based on scales of the two (Experiment 1) or three (Experiment 2) items.

Finally, in Experiment 2, we added a set of items designed to tap into willingness to act on behalf of each faction. Specifically, participants were asked: i) "If [treatment/focal faction] were to hold an event in your city or town, how likely is it that you would attend?", and ii) "If [treatment/focal faction] were to sponsor a petition, how likely is it that you would sign it?" Given high reliability (α = .84 for Treatment Faction and α = .84 for the Focal Faction), our analyses are based on a composite of the two items.

Primary ANOVA Analyses for Treatment Factions

Results from ANOVA analyses for the treatment factions are given in Tables S3 (Experiment1) and S4 (Experiment 2). Unlike the ANOVA for the focal factions (Tables 1 and 3 in the main text), our tactics and agenda treatments interact for some outcome measures related to the treatment faction. Given that our primary interest centers on the focal factions, we do not discuss all these interactions here. Instead, we simply note that a comparison of the condition means in Tables S3 and S4 shows that the effect of a radical agenda on support for the treatment faction was somewhat weaker when tactics were radical than when they were not.

Analyses of Moderation by Political Party Identification

We wanted to ensure that our key outcomes did not depend on participants' political identification. We therefore recruited roughly equal numbers of Republicans, Democrats and Independents using prescreen data from our panel. Specifically, panel participants responded to the following item adapted from the ANES (American National Election Studies): "Generally speaking do you think of yourself as a Republican, a Democrat, an Independent, or What?" We use participants' responses on this prescreen to code their political identification in our analyses.

For both studies, we created dummy variables for "Republican" and "Democrat." We replicated the analyses of Tables 1 and 3 (*Main Text*) for our primary outcome measures (identification, support and, for Experiment 2, willingness to act on behalf of the faction)

with these party identification dummies included in the models. As Table S5 demonstrates, in no case do we find any interaction between our treatments and political identification. Follow-up analyses show that Democrats were more likely to identify with and support the focal factions, while Republicans were less likely to identify with and support the focal factions. This is unsurprising given that the focal factions advocated for causes more aligned with Democratic priorities. But the absence of moderation effects across all key measures points to the robustness of the radical flank effects we observe.

Analysis of Non-Faction Items

It is important to know whether radical flanks increase support for the moderate faction's agenda without undermining support for broader movement issues, i.e., objectives beyond the agendas of the movement factions we investigated. To address this, participants in Experiment 1 were asked the following questions: To what extent do you think that animal rights and welfare is an important issue?, ii) How important is it that government agencies like the F.D.A. (Food and Drug Administration) improve enforcement of laws pertaining to factory farms?, iii) How important is it that Congress and government agencies work to create new laws and regulations to improve living conditions of animals in factory farms?, and iv) How likely is it that you will reduce your consumption of meat (e.g., by taking part in "Meatless Mondays") in the future? Our manipulations did not impact responses to any of these measures (independent sample t-tests; all *p*s > .10).

We also included a number of items in Experiment 2 that tracked outcomes other than those for the treatment and focal factions. Specifically, using items adapted from an environmental citizenship measure from (3), we asked participants how likely they would be to engage in the following activities in the next twelve months? 1. Sign a petition in support of environmental protection, 2. Call or write an elected representative to support environmental protection, 3. Vote for a candidate at least in part because he or she was in favor of environmental protection, 4. Give money to an environmental group. We also included several items adapted from the climate policy support measure from (4). Specifically, participants were asked to indicate the extent to which they oppose or support each of the following items: (0 = "Totally Oppose" to 100 = "Totally Support"). 1. Government regulations on industries and businesses that produce a great deal of greenhouse emissions linked to climate change, 2. Increasing taxes on industries and businesses that produce a lot of greenhouse emissions linked to climate change, 3. Providing a tax credit to people who do things to address climate change (for example, purchasing hybrid vehicles, purchasing energy efficient appliances, investing in home insulation). As in Study 1, we do not find any evidence that our manipulations (or their interaction) significantly impact responses on these items (independent sample t-tests; all *p*s > .10). These results strongly suggest that the presence of radical flanks boosts support and willingness to act on behalf of the focal group without harming more general support for the movement.

Robustness Checks for Key Outcomes

As a test of the robustness of our key findings linking the use of radical tactics by the treatment faction on support for – and willingness to act on behalf of – the focal faction, Table S6 presents results from linear regression models with an array of controls

for socio-demographics and political party identification. These models show that our key findings (Table 2 in the Main Text) are robust to model specification and the inclusion of an array of controls.

Sensitivity Analyses for Mediation Models

Here we report sensitivity analyses to assess the extent to which our key mediation analyses meet the sequential ignorability assumption (1). The first part of this assumption holds that, conditional on observed pre-treatment covariates, the treatment variable should be independent of potential values of the mediator and outcome variable. Our models satisfy this part of the assumption since we randomly assigned participants to the radical vs. moderate treatment faction conditions. The second part of the sequential ignorability assumption requires, conditional on observed treatment and pretreatment covariates, the mediator be independent of potential values of the outcome variable. Our sensitivity analyses assess the robustness of our mediation results to this second part of the sequential ignorability assumption, since participants were not randomly assigned to our mediator variables. Table S7 reports the results of sensitivity analyses using the Hicks and Tengley (2) stata package.

The righthand column shows, for the corresponding mediation result, the magnitude that the correlation (ρ) between the mediator's and dependent variable's (DV) errors would need to have in order to negate the mediation effect. The closer ρ is to 0, the more sensitive the mediation effect is to violations of the sequential ignorability assumption. The further from 0, the more robust it is to violations.

These sensitivity analyses suggest that those models with identification with the focal faction (where ρs $\geq .80$) are particularly robust to correlated errors. The models that include perceived normative support also appear to be robust, though less so (ρs $\geq .51$). The models involving perceived radicalness of the focal faction tactics, and particularly the model linking it to the outcome willingness to act on behalf of the focal faction (in Study 2) merit caution. These analyses show that even small correlated errors ($\rho = .11$) between the mediator and willingness to act outcome measure would be sufficient for our mediation model to be a false positive. That model should therefore be interpreted most cautiously. For the remaining models, sensitivity analyses suggest that our mediation effects are, to varying degrees, relatively robust.

Table S1. Measures of Perceived Radicalness of Tactics and Agenda

<table>
<tr><td></td><td></td><td></td><td></td></tr>
<tr><td colspan="2">Tactics Measures</td><td colspan="2">Agenda Measures</td></tr>
<tr><td>Experiment 1</td><td>Experiment 2</td><td>Experiment 1</td><td>Experiment 2</td></tr>
<tr><td colspan="2" rowspan="4">Extreme
Disruptive
Harmful
Unusual</td><td colspan="2">Extreme</td></tr>
<tr><td>Change to Status Quo</td><td></td></tr>
<tr><td></td><td>Radical</td></tr>
<tr><td></td><td>Disruptive</td></tr>
<tr><td>Normal or Typical (rev)</td><td>Radical</td><td></td><td>Harmful</td></tr>
</table>

Table S2: Measures of Perceived Normative Support

Tactics are Perceived as Normative		Agenda is Perceived as Normative	
Experiment 1	Experiment 2	Experiment 1	Experiment 2
	Would be supported by most people	Are supported by most people	Would be supported by most people
	Would generally be accepted by the broader public	Are generally accepted by the broader public	Would generally be accepted by the broader public

Table S3. Effect of Treatment Faction's Tactics (Moderate or Radical) and Agenda (Moderate or Radical) on Perceptions of and Support for the Treatment Faction. Experiment 1

	Means (SDs) by Condition				ANOVA Results		
	Mod. Tactics Mod. Agenda	Rad. Tactics Mod. Agenda	Mod. Tactics Rad. Agenda	Rad. Tactics Rad. Agenda	Radical Tactics	Radical Agenda	Interaction
Perceived Radicalness of Tactics	2.21 (1.09)	5.63 (1.18)	2.64 (1.41)	5.90 (1.09)	$F = 2164.22$ $p < .001$	$F = 24.07$ $p < .001$	$F = 1.13$ $p = .289$
Perceived Radicalness of Agenda	2.23 (1.40)	4.14 (2.15)	4.91 (2.13)	6.05 (1.33)	$F = 201.56$ $p < .001$	$F = 457.45$ $p < .001$	$F = 12.83$ $p < .001$
Identification with Treatment Faction	3.99 (1.77)	2.73 (1.71)	2.26 (1.50)	1.88 (1.38)	$F = 73.71$ $p < .001$	$F = 181.98$ $p < .001$	$F = 21.26$ $p < .001$
Support for Treatment Faction	5.10 (1.55)	3.81 (1.72)	2.84 (1.78)	2.21 (1.62)	$F = 91.95$ $p < .001$	$F = 371.41$ $p < .001$	$F = 11.06$ $p = .001$

Table S4. Effect of Treatment Faction's Tactics (Moderate or Radical) and Agenda (Moderate or Radical) on Perceptions of and Support for the Treatment Faction. Experiment 2.

	**Means (SDs) by Condition**				**ANOVA Results**		
	Mod. Tactics Mod. Agenda	Rad. Tactics Mod. Agenda	Mod. Agenda Rad. Tactics	Rad. Tactics Rad. Agenda	Radical Tactics	Radical Agenda	Interaction
Perceived Radicalness of Tactics	21.77 (20.90)	71.22 (22.48)	30.07 (24.99)	73.46 (20.61)	F = 1793.46 $p < .001$	F = 23.08 $p < .001$	F = 7.65 $p = .006$
Perceived Radicalness of Agenda	34.32 (25.82)	45.96 (29.00)	63.56 (27.92)	68.52 (27.23)	F = 37.68 $p < .001$	F = 367.18 $p < .001$	F = 6.10 $p = .014$
Identification with Treatment Faction	47.90 (30.67)	28.87 (29.88)	37.80 (28.97)	24.45 (26.74)	F = 128.23 $p < .001$	F = 25.80 $p < .001$	F = 3.93 $p = .048$
Support for Treatment Faction	66.28 (29.48)	41.78 (28.13)	53.00 (28.38)	34.47 (26.83)	F = 240.70 $p < .001$	F = 55.12 $p < .001$	F = 4.62 $p = .032$
Willingness to Act	46.03 (33.43)	28.95 (28.88)	36.29 (31.40)	23.87 (28.19)	F = 96.49 $p < .001$	F = 24.40 $p < .001$	F = 2.41 $p = .121$

Table S5. Political Affiliation Does Not Moderate any Key Outcome Measures (Compare to Tables 1 and 3 in Main Text).

	ANOVA Results: F values (p values)					
	Experiment 1		Experiment 2			
	Identify	Support	Perc. Norm. Support	Identify	Support	Willing to Act
Radical Tactics	10.27 (.001)	5.88 (.015)	26.31 (<.001)	12.99 (< .001)	9.78 (.002)	7.27 (.007)
Radical Agenda	5.20 (.023)	0.02 (.891)	1.12 (.289)	0.81 (.369)	0.07 (.788)	0.16 (.692)
Rep	5.06 (.025)	17.63 (< .001)	24.72 (<.001)	105.24 (.000)	177.38 (< .001)	90.73 (< .001)
Dem	7.01 (.008)	8.71 (.003)	16.09 (<.001)	78.35 (< .001)	67.87 (< .001)	93.08 (< .001)
Tactics x Agenda	.78 (.377)	2.27 (.132)	1.29 (.256)	0.24 (.623)	1.16 (.282)	0.00 (.953)
Rep x Tactics	0.18 (.675)	0.38 (.539)	.72 (.390)	.014 (.713)	0.07 (.788)	0.51 (.474)
Rep x Agenda	0.75 (.388)	0.68 (.409)	.27 (.606)	0.18 (.671)	0.05 (.821)	0.07 (.794)
Dem x Tactics	2.66 (.103)	2.05 (.153)	.10 (.748)	1.74 (.187)	0.71 (.400)	1.56 (.212)
Dem x Agenda	0.016 (.899)	0.02 (.966)	.14 (.707)	0.01 (.976)	0.01 (.941)	0.24 (.624)
Rep x Tac x Ag	0.862 (.353)	1.07 (.302)	.00 (.955)	0.07 (.787)	0.19 (.666)	1.14 (.285)
Dem x Tac x Ag	2.03 (.157)	1.01 (.315)	.01 (.919)	0.30 (.585)	0.72 (.396)	2.69 (.101)

Table S6. Standardized Coefficients for Key Outcome Variables with Demographic Controls, Experiment 1 (Animal Rights Movement) and Experiment 2 (Climate Movement)

	Support – Experiment 1	Support – Experiment 2	Willingness to Act – Experiment 2
Radical Tactics	.062*	.085***	.083***
Female	.162***	.101***	.099***
Year of Birth	.026	.105***	.100***
White	.004	-.065**	-.119***
Less Than HS	-.026	.025	.012
High School Degree	.007	-.044	-.040
Some College (< Bachelors)	.001	-.015	.010
Advanced Degree	.002	.011	-.007
Democrat	.072*	.178***	.216***
Republican	-.177***	-.314***	-.223***

*p ≤ .05; **p ≤ .01; ***p ≤ .001 For education, Bachelors' Degree is the reference category. For Political Party Identification, Independent is the reference category.

Table S7: Sensitivity Analyses for Mediation Models

	**Study 1**	ρ at which ACME=0
1	-> Perceived Radicalness of Focal Faction Tactics (mediator) -> Support for Focal Faction (DV)	-.41
2	-> Identification with Focal Faction (mediator) -> Support for Focal Faction (DV)	.80
	**Study 2**	ρ at which ACME=0
1	-> Perceived Radicalness of Focal Faction Tactics (mediator) -> Support for Focal Faction (DV)	-.35
2	-> Perceived Radicalness of Focal Faction Tactics (mediator) -> Willingness to Act on Behalf of Focal Faction (DV)	-.11
3	-> Identification with Focal Faction (mediator) -> Support for Focal Faction (DV)	.82
4	-> Identification with Focal Faction (mediator) -> Willingness to Act on Behalf of Focal Faction (DV)	.80
5	-> Perceived Normative Support for Focal Faction (mediator) -> Support for Focal Faction (DV)	.65
6	-> Perceived Normative Support for Focal Faction (mediator) -> Willingness to Act on Behalf of Focal Faction (DV)	.51

A

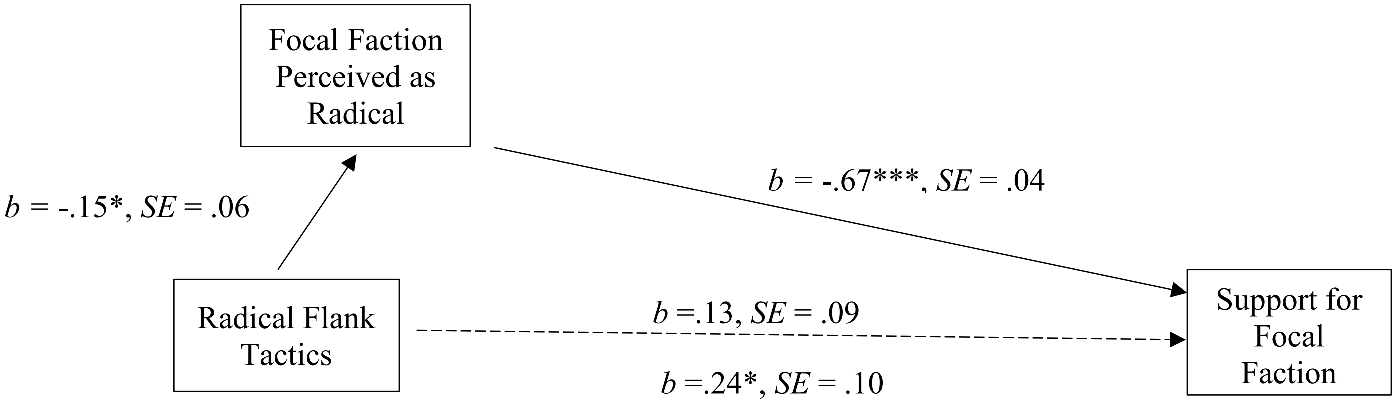

Focal Faction Perceived as Radical
b = -.15*, SE = .06
b = -.67***, SE = .04
Radical Flank Tactics
b =.13, SE = .09
b =.24*, SE = .10
Support for Focal Faction

B

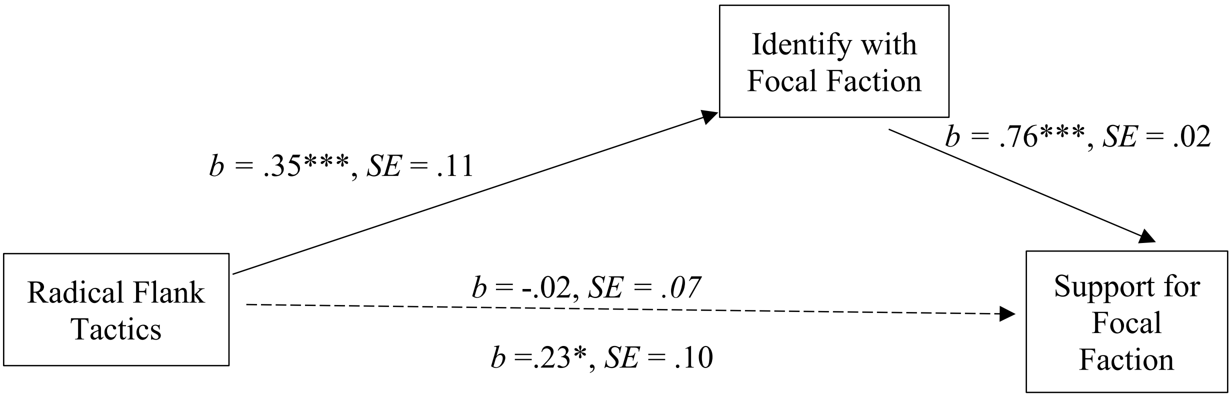

Identify with Focal Faction
b = .35***, SE = .11
b = .76***, SE = .02
Radical Flank Tactics
b = -.02, SE = .07
b =.23*, SE = .10
Support for Focal Faction

C

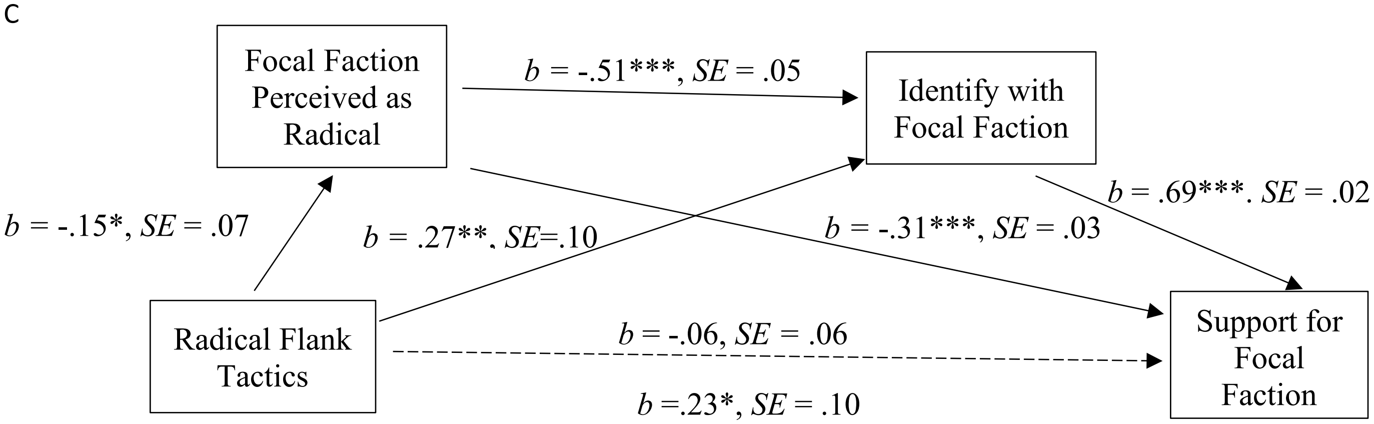

Fig. S1. Mediation Analyses for Support for Focal Faction, Experiment 1. Indirect Path: CI[.01, .19] (A). Indirect Path: CI[.11, .43] (B). Serial Path: CI[.01, .10] (C).
* $p \leq .05$; * $p \leq .01$; *** $p \leq .001$

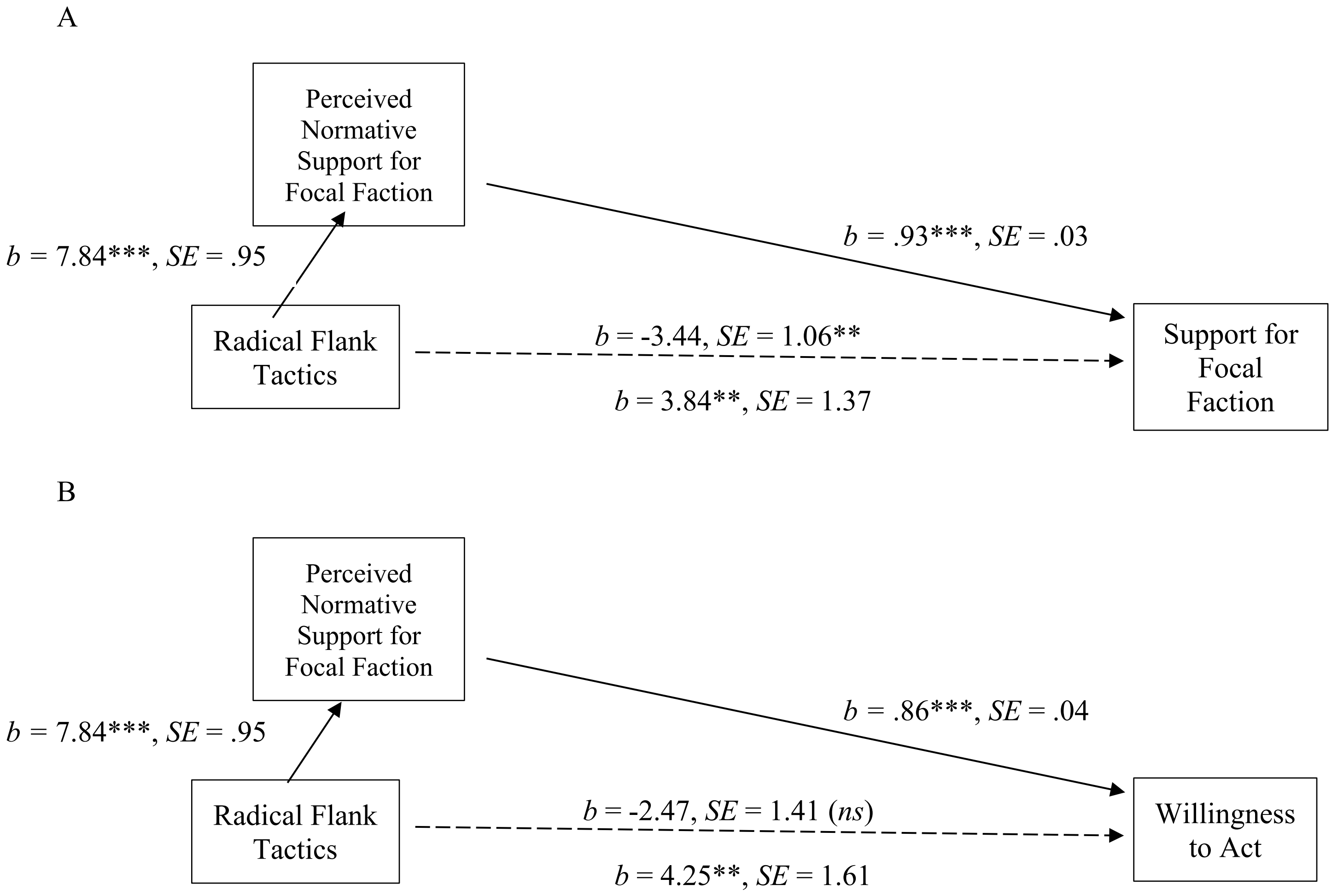

Fig. S2. Mediation Analyses with Perceived Normative Support as Mediator, Experiment 2
Support for the Focal Faction. Indirect Path: CI[5.50, 9.05] (A). *Willingness to Act on Behalf of the Focal Faction*. Indirect Path: CI[4.99, 8.51] (B).
* $p \leq .05$; ** $p \leq .01$; *** $p \leq .001$.

Full Text of Experimental Manipulations, Experiment 1

Condition: Radical Agenda/Radical Tactics
Objective No Animals For Food (NAFF) seeks to completely end human consumption of animals and the consumption or use of all animal byproducts (eggs, milk and other dairy, as well as leather).
NAFF was founded in 2011 to call attention to what they consider the needless consumption of animals by humans and the ineffectiveness of other organizations to eliminate animal consumption. Specifically, they argue that extreme cruelty is an inherent feature of animal consumption. By focusing government and citizens' attention on what they consider the immorality and injustice of eating animals and animal products, they aim to eliminate the human consumption of meat and thus to make the world a "Vegan World."
Activities: They have engaged in a range of activities to bring attention to their cause. They often stage protests outside meat production facilities and in large cities, where they block traffic or prevent entry into the offices of meat producers. NAFF demonstrators have doused streets and meat delivery trucks with the blood and entrails of animals slaughtered in factory farms in an effort to raise public awareness, and in some cases advocated violence against animal farmers.

Condition: Radical Agenda/Moderate Tactics
Objective No Animals For Food (NAFF) seeks to completely end human consumption of animals and the consumption or use of all animal byproducts (eggs, milk and other dairy, as well as leather).
NAFF was founded in 2011 to call attention to what they consider the needless consumption of animals by humans and the ineffectiveness of other organizations to eliminate animal consumption. Specifically, they argue that extreme cruelty is an inherent feature of animal consumption. By focusing government and citizens' attention on what they consider the immorality and injustice of eating animals and animal products, they aim to eliminate the human consumption of meat and thus to make the world a "Vegan World."
Activities: They have engaged in a range of activities to bring attention to their cause. They often stage demonstrations at universities, state capitals, and other public spaces. NAAF demonstrators often attract attention by organizing peaceful marches around cities where they carry signs and sing songs. They also provide passersby with literature detailing abusive factory farm practices, and organize "teach ins" to educate the public about veganism.

Condition: Moderate Agenda/Radical Tactics
Objective: Americans Against Animal Cruelty (AAAC) seeks to improve the conditions in factory farms while also encouraging Americans to reduce their meat consumption and to buy meat and meat products from "certified cruelty free" farms.
AAAC started in 2011 to call attention to what they consider the unnecessarily inhumane treatment of animals raised in factory farms and meat facilities. Specifically, they argue that extreme cruelty is not an inherent feature of meat production and thus advocate for more humane meat production practices. By focusing government and citizens' attention

on the practices of factory farms, they aim to increase the number of farms that are using humane methods to produce meat and thus to make the world a “Cruelty Free World.”
Activities: They have engaged in a range of activities to bring attention to their cause. They often stage protests outside meat production facilities and in large cities, where they block traffic or prevent entry into the offices of meat producers. AAAC demonstrators have doused streets and meat delivery trucks with the blood and entrails of animals slaughtered in factory farms in an effort to raise public awareness, and in some cases advocated violence against owners of factory farms.

Condition: Moderate Agenda/Moderate Tactics
Objective: Americans Against Animal Cruelty (AAAC) seeks to improve the conditions in factory farms while also encouraging Americans to reduce their meat consumption and to buy meat and meat products from “certified cruelty free” farms.
AAAC started in 2011 to call attention to what they consider the unnecessarily inhumane treatment of animals raised in factory farms and meat facilities. Specifically, they argue that extreme cruelty is not an inherent feature of meat production and thus advocate for more humane meat production practices. By focusing government and citizens’ attention on the practices of factory farms, they aim to increase the number of farms that are using humane methods to produce meat and thus to make the world a “Cruelty Free World.”
Activities: They have engaged in a range of activities to bring attention to their cause. They often stage demonstrations at universities, state capitals, and other public spaces. AAAC demonstrators often attract attention by organizing peaceful marches around cities where they carry signs and sing songs. They also provide passersby with literature detailing abusive factory farm practices, and organize “teach ins” to educate the public about conditions in factory farms.

Full Text of Focal Faction, Experiment 1

People Against Cruelty to Animals (PACA)
Objective: The primary objective of People Against Cruelty to Animals (PACA) is to improve the conditions of animals raised for food.
Established in 2007, PACA aims to raise public awareness of what they view as inhumane treatment of animals raised for food. They seek to draw attention to the cramped cages of chickens and the inability of cows raised for meat or dairy to graze outdoors. By raising public awareness of these conditions, they hope to pressure Congress and government agencies (such as the F.D.A.) to establish laws and regulations that will lead to more favorable living conditions for animals raised for food, such as larger pens and more time for animals to graze outdoors.
Activities: To realize these goals, the group stages demonstrations outside factory farms, the headquarters of global meat producers and in public spaces. These demonstrations generally involve singing and chanting, and speakers who discuss current living conditions of animals raised for food and how citizens can put pressure on their representatives to change these conditions.

Full Text of Manipulations, Experiment 2

Condition: Radical Agenda/Radical Tactics

Climate Action Today seeks an immediate end to the use of all fossil fuels, including fuel for automobiles and homes and other buildings, within a year. After one year, the group aims to have all existing cars and heating systems decommissioned if they cannot be retrofitted to run on renewal energy. The group's website states that while this plan will lead to a large disruption in daily life, this is precisely the point. "Only by stopping the use of fossil fuels dead in its tracks will there be the necessary public outrage and political will for transition to renewal energies, including solar and wind power, and a permanent solution to the existential threat of climate change." They have engaged in a range of activities to bring attention to their cause. They often stage protests outside the headquarters of fossil fuel companies, where they prevent entry into the offices of the companies. **Climate Action Today** demonstrators have defaced the exterior of the buildings containing the headquarters of fossil fuel companies, including breaking doors and windows, and spray-painting the words "liars" and "shame" on windows and walls. At one recent event, organizers threw stones and bottles at the cars of employees of fossil fuel company.

Condition: Radical Agenda/Moderate Tactics

Climate Action Today seeks an immediate end to the use of all fossil fuels, including fuel for automobiles and homes and other buildings, within a year. After one year, the group aims to have all existing cars and heating systems decommissioned if they cannot be retrofitted to run on renewal energy. The group's website states that while this plan will lead to a large disruption in daily life, this is precisely the point. "Only by stopping the use of fossil fuels dead in its tracks will there be the necessary public outrage and political will for transition to renewal energies, including solar and wind power, and a permanent solution to the existential threat of climate change." They have engaged in a range of activities to bring attention to their cause. They often stage demonstrations in public spaces. **Climate Action Today** demonstrators attract attention by organizing peaceful marches around cities where they carry signs and sing songs. They provide passersby with literature explaining, in simple terms, both the scientific consensus about climate change and steps they believe that must be taken to halt it. They also sponsor mass "teach-ins" to educate the public about climate change and the need to address it.

Condition: Moderate Agenda/Radical Tactics

Climate Action Today seeks a phasing out of the use of fossil fuels for automobiles and heating systems in homes and other buildings over the next 15 years. Over the course of these 15 years, existing cars and heat for homes and other buildings that rely on fossil fuels would be phased out as new cars and heating systems using renewable energy are phased in. The group states that the transition will be made easy via increased research and subsides on alternatives to fossil fuels, including solar and wind power. Further, the group's website states: "By having a clear and feasible transition period, we can minimize the disruption to daily life and permanently solve the existential threat of climate change problems." They have engaged in a range of activities to bring attention to their cause. They often stage protests outside the headquarters of fossil fuel

companies, where they prevent entry into the offices of the companies. **Climate Action Today** demonstrators have defaced the exterior of the buildings containing the headquarters of fossil fuel companies, including breaking doors and windows, and spray-painting the words “liars” and “shame” on windows and walls. At one recent event, organizers threw stones and bottles at the cars of employees of fossil fuel company.

Condition: Moderate Agenda/Moderate Tactics
Climate Action Today seeks a phasing out of the use of fossil fuels for automobiles and heating systems in homes and other buildings over the next 15 years. Over the course of these 15 years, existing cars and heat for homes and other buildings that rely on fossil fuels would be phased out as new cars and heating systems using renewable energy are phased in. The group states that the transition will be made easy via increased research and subsides on alternatives to fossil fuels, including solar and wind power. Further, the group’s website states: “By having a clear and feasible transition period, we can minimize the disruption to daily life and permanently solve the existential threat of climate change problems.” They have engaged in a range of activities to bring attention to their cause. They often stage demonstrations in public spaces. **Climate Action Today** demonstrators attract attention by organizing peaceful marches around cities where they carry signs and sing songs. They provide passersby with literature explaining, in simple terms, both the scientific consensus about climate change and steps they believe that must be taken to halt it. They also sponsor mass “teach-ins” to educate the public about climate change and the need to address it.

Full Text of Focal Faction, Experiment 2
Global Warming Warning is focused on educating the public about the scientific consensus about the human activities causing global warming and climate change, communicating the urgency of addressing climate change, and identifying global solutions to climate change. In developing and advocating for such solutions, they use scientific research to develop “best practices” for specific ecosystems and communities of the world. For instance, in highly industrialized communities, they focus more on incentivizing cleaner energies for buildings and transportation. For less industrialized contexts, they instead focus on the maintenance and preservation of large forests.
They have engaged in a range of activities to bring attention to their cause. They lobby state and national governments, as well as global leaders, such as the World Health Organization. They often stage peaceful demonstrations in public spaces, especially in front of government agencies and other high traffic areas. They also sponsor petitions to local and national government agencies advocating for green policies, including clean energy incentives and the preservation of forests and water systems. Finally, they sponsor local training programs to educate local stakeholders about how to best manage and improve infrastructure and natural resources.

SI References

1. K. Imai, L. Keele, D. Tingley, A general approach to causal mediation analysis. *Psychological methods* **15**, 309 (2010).
2. R. Hicks, D. Tingley, Causal mediation analysis. *The Stata Journal* **11**, 605–619 (2011).
3. P. G. Bain, M. J. Hornsey, R. Bongiorno, Y. Kashima, C. R. Crimston, Collective futures: How projections about the future of society are related to actions and attitudes supporting social change. *Personality and Social Psychology Bulletin* **39**, 523–539 (2013).
4. R. Bayes, J. N. Druckman, A. Goods, D. C. Molden, When and how different motives can drive motivated political reasoning. *Political Psychology* **41**, 1031–1052 (2020).
