# Supplementary material for: Radical flanks of social movements can increase support for moderate factions
Source: PNAS Nexus. 2022 Aug 4;1(3):pgac110. doi: 10.1093/pnasnexus/pgac110 (PMC9896934; doi:10.1093/pnasnexus/pgac110)
Supplement: pgac110_Supplemental_Files [file pgac110_supplemental_files.zip › PNASNEXUS-PNASNEXUS-2021-00138-s02.pdf]

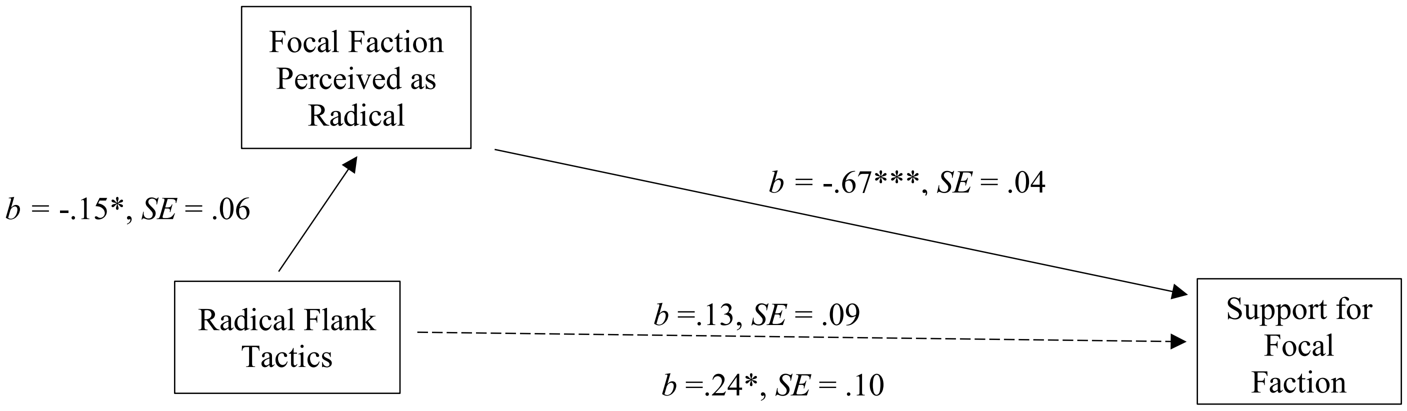

Fig. S1a. Mediation model for *Support for the Focal Faction*.
* $p \leq .05$; ** $p \leq .01$; *** $p \leq .001$
Indirect Path: CI[.01, .19]

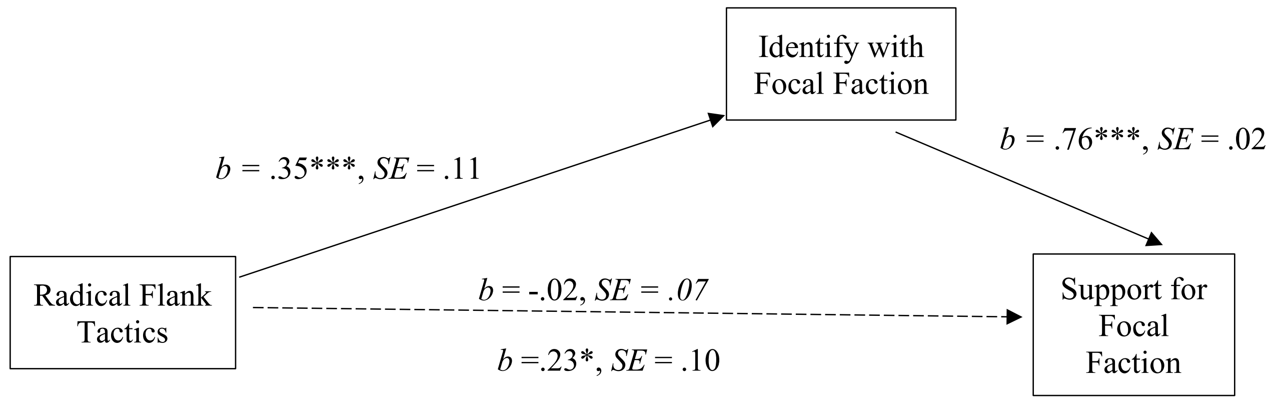

Fig. S1b: Mediation model for *Support for the Focal Faction*.
* $p \leq .05$; ** $p \leq .01$; *** $p \leq .001$
Indirect Path: CI[.11, .43]

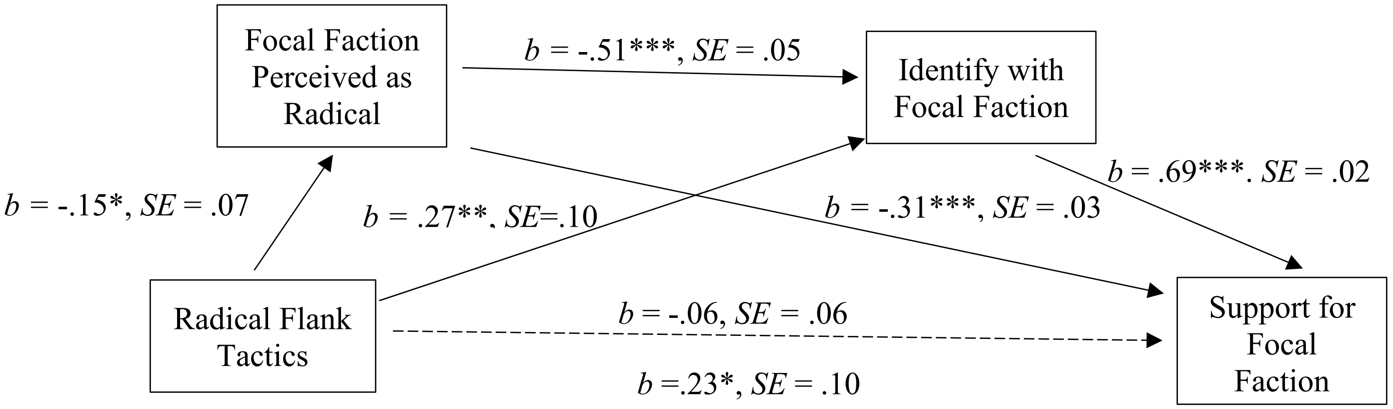

Fig. S1c. Serial mediation model for *Support for the Focal Faction*.
* $p \leq .05$; * $p \leq .01$; *** $p \leq .001$
Serial Path: CI[.01, .10]

Fig. S1. Mediation Analyses for Support for Focal Faction, Experiment 1

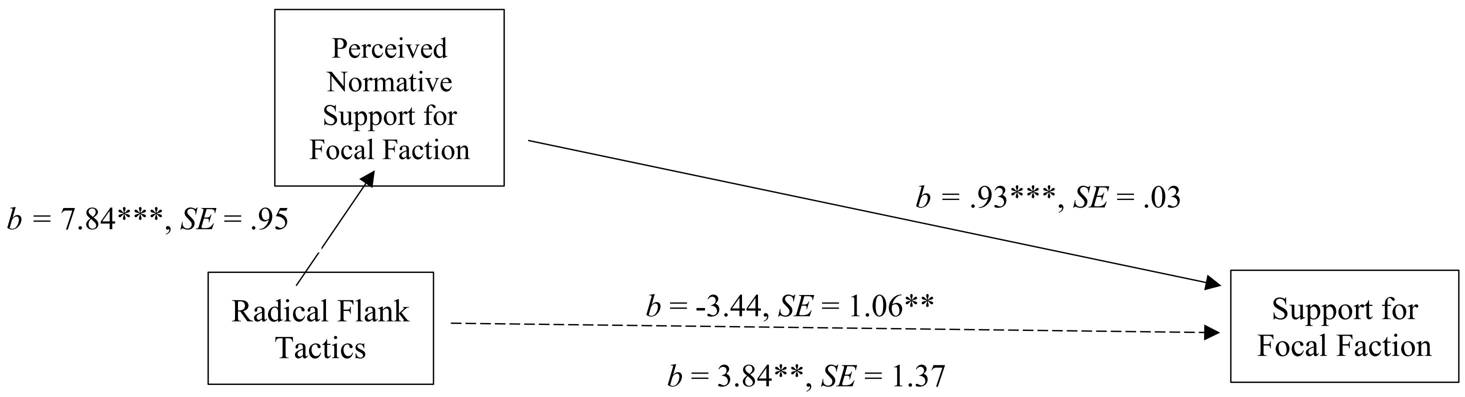

Figure 2a. Mediation model for *Support for the Focal Faction*.
* $p \leq .05$; ** $p \leq .01$; *** $p \leq .001$.
Indirect Path: CI[5.50, 9.05]

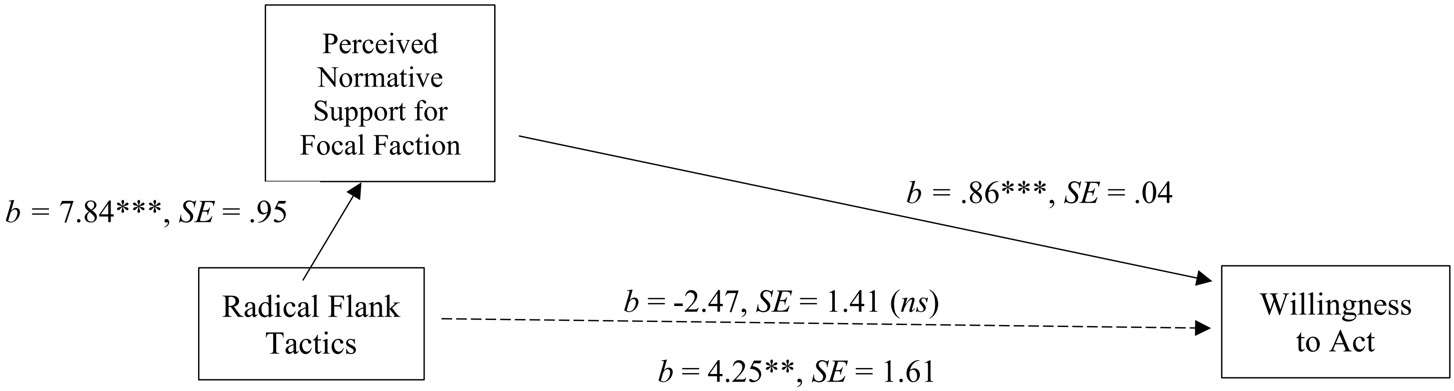

Figure 2b. Mediation model for *Willingness to Act on Behalf of the Focal Faction*.
* $p \leq .05$; ** $p \leq .01$; *** $p \leq .001$.
Indirect Path: CI[4.99, 8.51]
